# Modeling pathway overlap increases accuracy of GWAS gene set enrichment

**DOI:** 10.64898/2026.09.03.26362206

**Published:** 2026-09-07

**Authors:** Alanna C. Cote, Reagan Kesting, Judit García-González, Paul F. O’Reilly

## Abstract

Genome-wide association studies (GWAS) have identified thousands of loci associated with complex traits and diseases, and extensive efforts are underway to translate these variant-level signals into biological mechanism. A widely applied approach is pathway enrichment analysis, which tests whether genetic associations concentrate within biological pathways beyond background polygenic expectations. However, pathway databases contain extensive sharing of genes across pathways (“pathway overlap”), an underappreciated source of bias that creates structural dependencies in enrichment statistics and obscures pathway-specific genetic signal. Moreover, the degree of pathway overlap is increasing as pathway resources expand. Here, we introduce Gene Swap Randomization (GSR), an empirical framework that preserves pathway size and multi- pathway gene membership in the null model, enabling explicit adjustment for pathway overlap. Applying GSR to enrichment results from the Molecular Signatures Database (MSigDB) across twelve complex traits and four pathway analysis approaches (MAGMA, PascalX, GSA-MiXeR, and PRSet), we show that pathway overlap can produce enrichment under polygenicity even in the absence of pathway-specific biology. GSR improves prioritization of biologically relevant pathways supported by independent gene-disease associations (Open Targets, Malacards), regulatory interactions (DoRothEA), and tissue-specific expression patterns (GTEx). GSR improves concordance with external benchmarks in 60.8% of comparisons overall and 79.3% disease- association benchmarks, corresponding to improvement in 10 of 16 aggregated method-validation framework comparisons. We demonstrate that pathway overlap is a key source of bias in GWAS pathway enrichment, that pathway-specific disease enrichment persists after conditioning on overlap, and that GSR improves biological insight by distinguishing pathway-specific genetic signal from enrichment driven by pathway overlap.

## Full Text Availability

The license terms selected by the author(s) for this preprint version do not permit archiving in PMC. The full text is available from the preprint server.

